# Disease burden due to biomass cooking-fuel-related household air pollution among women in India

**DOI:** 10.3402/gha.v7.25326

**Published:** 2014-11-04

**Authors:** Meena Sehgal, Suliankatchi Abdulkader Rizwan, Anand Krishnan

**Affiliations:** 1 Centre for Environmental Studies, The Energy and Resources Institute, New Delhi, India; 2 Velammal Medical College Hospital & Research Institute, Tamil Nadu, India; 3 Centre for Community Medicine, All India Institute of Medical Sciences, New Delhi, India

**Keywords:** household air pollution, biomass fuel, population attributable fraction

## Abstract

**Background:**

Household air pollution (HAP) due to biomass cooking fuel use is an important risk factor for a range of diseases, especially among adult women who are primary cooks, in India. About 80% of rural households in India use biomass fuel for cooking. The aim of this study is to estimate the attributable cases (AC) for four major diseases/conditions associated with biomass cooking fuel use among adult Indian women.

**Methods:**

We used the population attributable fraction (PAF) method to calculate the AC of chronic bronchitis, tuberculosis (TB), cataract, and stillbirths due to exposure to biomass cooking fuel. A number of data sources were accessed to obtain population totals and disease prevalence rates. A meta-analysis was conducted to obtain adjusted pooled odds ratios (ORs) for strength of association. Using this, PAF and AC were calculated using a standard formula. Results were presented as number of AC and 95% confidence intervals (CI).

**Results:**

The fixed effects pooled OR obtained from the meta-analysis were 2.37 (95% CI: 1.59, 3.54) for chronic bronchitis, 2.33 (1.65, 3.28) for TB, 2.16 (1.42, 3.26) for cataract, and 1.26 (1.12, 1.43) for stillbirths. PAF varied across conditions being maximum (53%) for chronic bronchitis in rural areas and least (1%) for cataract in older age and urban areas. About 2.4 (95% CI: 1.4, 3.1) of 5.6 m cases of chronic bronchitis, 0.3 (0.2, 0.4) of 0.76 m cases of TB, 5.0 (2.8, 6.7) of 51.4 m cases of cataract among adult Indian women and 0.02 (0.01, 0.03) of 0.15 m stillbirths across India are attributable to HAP due to biomass cooking fuel. These estimates should be cautiously interpreted in the light of limitations discussed which relate to exposure assessment, exposure characterization, and age-specific prevalence of disease.

**Conclusions:**

HAP due to biomass fuel has diverse and major impacts on women’s health in India. Although challenging, incorporating the agenda of universal clean fuel access or cleaner technology within the broader framework of rural development will go a long way in reducing disease burden.

Household air pollution (HAP) due to solid fuel use is considered the fourth leading risk factor of global disease burden and the third leading risk factor in India (1). Solid fuel or biomass fuel refers to the use of cheap materials such as wood, crop residues, or cow dung for cooking or heating purposes, mainly in the poorer regions of the world.

Biomass fuel remains a widely used energy source in rural India where nearly 80% of households use them as the primary cooking fuel. In contrast, the majority of urban households use liquefied petroleum gas (LPG) as the primary cooking fuel; however, about 19% of urban households use biomass fuel for cooking purposes. Poverty, inaccessibility to improved cooking fuel, and lack of awareness about harms of biomass emissions are among the major factors that drive their widespread use (2). Use of biomass fuel leads to harmful health effects due to the emission, during its incomplete combustion, of a large number of air pollutants such as carbon monoxide (CO), sulfur dioxide (SO_2_), respirable particulate matter (PM_2.5_ and PM_10_), poly-cyclic aromatic hydrocarbon (PAH), benzene, and metals like lead and copper.

Chronic exposure to biomass smoke-generated indoors can cause a wide range of health effects such as chronic obstructive pulmonary disease (COPD) (3–5), tuberculosis (TB) (6–9), cataract (10–12), and adverse pregnancy outcomes such as stillbirths, low birth weight, intrauterine growth retardation, and infant mortality (13–15).

Despite the availability of ample evidence on the adverse consequences of biomass fuel use in India, the national response to the issue has been limited. This is partly because of limited access and awareness among researchers and partly due to the fact that this evidence has not been packaged into the language of policy makers. India has recently identified HAP as one of the key indicators in its National Monitoring Framework for Prevention and Control of Non-Communicable Diseases (NCD) (16). This provides an excellent opportunity to advocate the reduction of health effects due to household biomass fuel use.

Although a handful of Indian studies have established association between HAP due to biomass fuel and specific disease conditions, few have considered attributable fractions which are a more policy-oriented way of expressing the burden. Also, there is a lack of studies which provide comprehensive estimates for a range of conditions caused by biomass-associated HAP. Generating evidence-based burden of disease estimates due to biomass-related HAP would help policy makers appreciate the seriousness of the problem and accord it due priority among other competing issues. Such estimates were recently generated for under-five children (17). This paper aims to generate burden estimates of a range of disease conditions attributable due to biomass-related HAP among adult rural and urban Indian women because they form a large part of the exposed group.

## Methodology

### Data sources

In order to arrive at a comprehensive estimate of disease burden due to biomass cooking-fuel-related HAP, we needed to access a number of data sources. The reference year for this study was 2011. An attempt was made to ensure that all available data were as close to 2011 as possible. Primary exposure variable was use of biomass fuel for cooking purposes. We considered disease burden due to four conditions (chronic bronchitis, TB, cataract, and stillbirths) as primary outcomes. These diseases were chosen as the evidence of association was best for them and also because they represented diseases in different life stages. We used the population attributable fraction (PAF) method to calculate estimates of disease burden (18).

#### Exposure data

Exposure data were obtained from the National Sample Survey Office (NSSO) report. The NSSO conducts nationwide yearly surveys, which serve as reliable sources of information on specific themes such as household consumer expenditure, food consumption, employment, drinking water, sanitation, and housing conditions at the national level. In 2009–10, the 66th round of NSS collected information on energy sources of Indian households for cooking and lighting, from which we calculated the prevalence of biomass fuel used for cooking purposes (defined here as the use of firewood, crop residue, or dung cake as the primary source of cooking fuel). We did not include biomass fuel used for heating or lighting purposes in this study because their emission levels, exposure, and health risks are likely to be different from cooking fuel. Detailed methodology of the survey is given elsewhere, but briefly the NSS 66th round was a stratified multistage survey covering the whole of India with non-institutionalized usual residents as the eligible population. A total of 59,119 rural and 41,736 urban households were sampled in this survey (2).

#### Outcomes data

Total number of women in the country by residence and age was obtained from the primary census abstract data and single-year age data of Census 2011, provided by the Office of the Registrar General and Census Commissioner of India (ORGCMI). Census of India, a decennial exercise of complete enumeration of all the people in the country, was last conducted in 2011 (19).

Crude Birth Rates (CBR) and Stillbirth Rates (SBR) by residence for the year 2011 were obtained from the Sample Registration System (SRS) report provided by the ORGCMI (20). The SRS survey, a unistage stratified simple random survey, is a continuous enumeration process conducted all year round in selected sample units spread across the country and serves as an important source of state-wise fertility and mortality indicators. The 2011 survey included 4,433 rural and 3,164 urban sampling units covering a population of 7,352,000.

Prevalence rates of disease conditions were obtained from country-level or large representative published studies. An attempt was made to obtain rates that were closest to 2011, by residence, gender, and age. Age groups were decided on the basis of availability of information and natural history of disease (age at exposure and time to disease). Strength of association parameters (either odds ratios [ORs] or relative risks [RRs]) between biomass fuel exposure and disease/condition were obtained through a systematic review and meta-analysis procedure (see Supplementary Fig. 1 and Supplementary Table 1a–c).In brief, studies included for meta-analysis were either undertaken in India or in their absence in South-East Asia, these studies included biomass cooking fuel use as exposure, any one of the stated conditions as outcome, included either non-smoking adult women as study population or provided OR/RR either adjusted or stratified by important confounding variables such as smoking. A fixed effects meta-analysis was conducted on the adjusted OR/RR provided by the studies to obtain pooled estimates (Supplementary Table 1).

### Estimation procedure

We have used the PAF method to calculate the total number of cases of the four stated conditions attributable to biomass cooking fuel exposure. We used the formula given by Rothman and Greenland (21) for PAF calculation.

$$\text{PAF}=\frac{{\text{P}}_{\text{e}}(\text{OR}-1)}{{\text{P}}_{\text{e}}(\text{OR}-1)+1}$$

where PAF is population attributable fraction, Pe is proportion of population exposed to biomass cooking fuel (primary study exposure), and OR is adjusted odds ratio (calculated from meta-analysis).

The total number of attributable cases (AC) of a particular condition was then obtained by the formula:

AC=PAF×TC

where AC is attributable cases of a particular condition (primary study outcome), PAF is population attributable fraction, and TC is total number of cases of a particular condition (obtained by multiplying residence, gender- and age-specific prevalence rates to the corresponding national women population and summing up the category totals).

### Statistical analysis

All data were entered in Microsoft Excel and calculations were made by incorporating the above formula. Wherever needed, 95% confidence intervals (CI) were provided. We used Comprehensive Meta-Analysis version 2.2 (Biostat, Englewood, NJ) for conducting fixed effects meta-analysis. The effect size of interest was OR of association between biomass cooking fuel use and the particular disease/condition. Heterogeneity between studies was examined using I square and Cochran’s Q statistics.

## Results

In this study, it was not possible to obtain age-, gender-, and residence-wise data for all possible scenarios, hence several assumptions had to be made (Box 1).

*Box 1*. Assumptions made in the estimation procedure

***Exposure –*** **biomass cooking fuel use**

Prevalence of biomass exposure is the same across different age groups (above 25 years) of women.

Toxic constituents of different types of biomass fuel have similar health effects.

***Outcome***

**Chronic bronchitis**: Age-specific prevalence is the same across residence. Some studies used to calculate strength of association (OR) between chronic bronchitis and biomass cooking fuel exposure did not include chronic bronchitis as the outcome per se, therefore chronic obstructive pulmonary disease (COPD) or abnormal peak expiratory flow rate (PEFR) are taken to serve as proxy.

OR and consequently PAF are the same across age groups and residence.

Age group at risk – 35 years and above.

**Tuberculosis**: Since prevalence for pulmonary TB alone was not available for the year 2011, prevalence of all TB cases (smear negative pulmonary, smear positive pulmonary and extra-pulmonary) for the year 2011 is taken to be the same as pulmonary TB.

Prevalence is the same across gender, age groups and residence. OR is the same across age groups and residence, and PAF is the same across age groups.

Age group at risk – 25 years and above.

**Cataract**: Prevalence is the same across residence. Prevalence of disease in the unexposed population (Po) required for conversion of OR to RR is assumed to be approximately equal to general population prevalence.

Age group at risk – 50***–***69 years.

**Stillbirth**: There are two sources of stillbirth rate. The SRS report of 2011 gave estimates for the year 2011 and Cousens et al. reported estimates for the year 2009. We used both sources.

### Exposure assessment

#### Biomass cooking fuel use

Prevalence of biomass fuel used for cooking purposes was calculated by adding up the proportion of households that reported use of firewood, chips, and dung cake as cooking fuel. It was seen that 82.6% of rural and 18.8% of urban households used biomass fuel as the primary source of cooking fuel (Table 1).

**Table 1:** Key input parameters for exposure and outcome assessment

	Proportion		Number of cases		
	
Parameter for women age over 25 years	Rural	Urban	Rural	Urban	Sources
**Exposure**, % (age-specific rates – NA)					
Prevalence of firewood and chips as cooking fuel	76.3	17.5	151,102,748	17,251,243	NSSO report, 2009–10 (data for 2009) (2)
Prevalence of dung cake as cooking fuel	6.3	1.3	12,476,374	1,281,521
Prevalence of total biomass cooking fuel use	82.6	18.8	163,579,122	18,532,764
**Outcome**					
Population totals for adult women					
25–34	65.6	34.4	61,647,268	32,334,166	Census, 2011 (data for 2011) (19)
35–44	66.1	33.9	50,937,056	26,161,110
45–54	65.7	34.3	35,054,663	18,340,002
55–64	69.0	31.0	26,678,850	11,967,363
65–74	71.7	28.3	16,535,168	6,530,620	
≥ 75	68.9	31.1	7,184,673	3,245,269	
Total	66.8	33.2	198,037,678	98,578,530	
Prevalence of chronic bronchitis, % (residence wise rates – NA)					
35–44	1.33		676,986	347,698	Jindal et al. (data for 2009) (3)
45–54	2.55		895,288	468,400
55–64	3.43		916,202	410,982	
65–74	5.42		895,380	353,633	
≥ 75	6.25		449,305	202,948	
Total			3,833,161	1,783,660	
Prevalence of TB (per 100,000 population, residence wise and age-specific rates – NA)	256		506,976	252,361	RNTCP annual status report, 2013 (data for 2011) (22)
Prevalence of cataract, % (residence wise rates – NA)					
50–59	54.5		15,569,138	7,820,099	Murthy et al. (data for 2002–03) (23)
60–69	86.3		19,907,349	8,116,518
Total			35,476,487	15,936,616
Crude birth rate (per 1,000 population)	23.3	17.6	19,342,347	6,605,834	SRS report, 2011 (data for 2011) (20)
Stillbirth rate (per 1,000 live births)					
Source 1	6	6	116,054	39,635	SRS report, 2011 (data for 2011) (20)
Source 2	22	22	425,532	145,328	Cousens et al., (data for 2009) (26)
Strength of association from meta-analysis (residence wise and age-specific rates – NA)					
Chronic bronchitis, pooled OR (95% CI)	2.37 (1.59, 3.54)				Johnson et al. (4); Sukhsohale et al. (27)
Tuberculosis, pooled OR (95% CI)	2.33 (1.65, 3.28)				Behera and Aggarwal (6); Lakshmi et al. (7); Mishra et al. (9)
Cataract, pooled OR (95% CI)	2.16 (1.42, 3.26)				Zodpey and Ughade (10); Pokhrel et al. (11)
Cataract^a^ – 50–59 years, pooled RR (95% CI)	1.32 (1.16, 1.46)				Zodpey and Ughade (10); Pokhrel et al. (11)
Cataract^a^ – 55–69 years, pooled RR (95% CI)	1.08 (1.04, 1.11)				Zodpey and Ughade (10); Pokhrel et al. (11)
Stillbirth, pooled OR (95% CI)	1.26 (1.12, 1.43)				Lakshmi et al. (13); Mishra et al. (14)

a RR was calculated from OR approximation formula because cataract was not a rare disease. NA=not available.

### Outcome assessment

#### Chronic bronchitis

There were no national-level survey-based estimates of chronic bronchitis available in India. The latest available large-scale prevalence data for chronic bronchitis came from a multi-centric study undertaken in 2009 across 12 Indian cities with a sample that included 84,470 women (3). It employed a two-stage, stratified sampling design and included people aged ≥15 years. The study provided 10-year age-specific prevalence rates 35 years onward. Prevalence increased with age, reaching a maximum of 6.25% in the ≥75 age group. By applying these rates to the age-specific national women population, we estimated the total number of chronic bronchitis cases, which was 3,833,161 in rural and 1,783,660 in urban areas (Table 1). OR calculated from the meta-analysis of two studies showed that chronic bronchitis was nearly two times more common among women exposed to biomass cooking fuel (OR = 2.37, 95% CI: 1.59, 3.54) (Supplementary Table 1b). About 53% of cases (PAF = 0.53, 95% CI: 0.33, 0.68) in rural and 20% (PAF = 0.20, 95% CI: 0.10, 0.32) in urban areas could be attributed to biomass cooking fuel, that is, about 2.4 (95% CI: 1.4, 3.2) out of the 5.6 m cases in the country (Table 2 and Supplementary Table 2).

**Table 2:** Biomass cooking-fuel-related PAF and AC of specific conditions

	PAF		AC								
	
	Rural	Urban	Rural			Urban			Total		
	
Condition	Estimate (95% CI)		Estimate	Lower CI	Upper CI	Estimate	Lower CI	Upper CI	Estimate	Lower CI	Upper CI
Chronic bronchitis											
Age (years), 35–44	0.53 (0.33, 0.68)	0.20 (0.10, 0.32)	359,394	221,820	458,465	71,212	34,716	112,373	430,606	256,536	570,838
45–54	0.53 (0.33, 0.68)	0.20 (0.10, 0.32)	475,285	293,349	606,303	95,933	46,767	151,382	571,217	340,117	757,685
55–64	0.53 (0.33, 0.68)	0.20 (0.10, 0.32)	486,387	300,202	620,466	84,173	41,035	132,825	570,560	341,236	753,291
65–74	0.53 (0.33, 0.68)	0.20 (0.10, 0.32)	475,333	293,379	606,365	72,427	35,309	114,291	547,760	328,688	720,656
≥75	0.53 (0.33, 0.68)	0.20 (0.10, 0.32)	238,524	147,219	304,276	41,566	20,263	65,591	280,089	167,482	369,867
Total			2,034,922	1,255,969	2,595,875	365,310	178,090	576,462	2,400,233	1,434,059	3,172,337
Tuberculosis											
Age (years), 25–34	0.52 (0.35, 0.65)	0.20 (0.11, 0.30)	82,615	55,132	103,082	16,557	9,014	24,835	99,172	64,145	127,917
35–44	0.52 (0.35, 0.65)	0.20 (0.11, 0.30)	68,262	45,553	85,173	13,396	7,293	20,094	81,658	52,846	105,267
45–54	0.52 (0.35, 0.65)	0.20 (0.11, 0.30)	46,978	31,350	58,616	9,391	5,113	14,087	56,369	36,462	72,702
55–64	0.52 (0.35, 0.65)	0.20 (0.11, 0.30)	35,753	23,859	44,610	6,128	3,336	9,192	41,881	27,195	53,802
65–74	0.52 (0.35, 0.65)	0.20 (0.11, 0.30)	22,159	14,788	27,649	3,344	1,821	5,016	25,503	16,608	32,665
≥75	0.52 (0.35, 0.65)	0.20 (0.11, 0.30)	9,628	6,425	12,014	1,662	905	2,493	11,290	7,330	14,506
Total			265,396	177,107	331,143	50,479	27,480	75,717	315,874	204,587	406,860
Cataract											
Age (years), 50–59	0.21 (0.12, 0.28)	0.06 (0.03, 0.08)	3,281,930	1,802,405	4,298,169	448,162	226,287	624,546	3,730,092	2,028,692	4,922,714
60–69	0.06 (0.03, 0.08)	0.01 (0.01, 0.02)	1,225,502	682,293	1,590,153	119,400	65,036	157,264	1,344,902	747,329	1,747,418
Total			4,507,432	2,484,698	5,888,322	567,563	291,323	781,810	5,074,994	2,776,021	6,670,132
Stillbirth											
@ 6/1,000 LB	0.18 (0.09, 0.26)	0.05 (0.02, 0.07)	20,517	10,466	30,417	1,847	874	2,964	22,365	11,340	33,381
@ 22/1,000 LB	0.18 (0.09, 0.26)	0.05 (0.02, 0.07)	75,231	38,375	111,528	6,773	3,206	10,870	82,003	41,581	122,397

PAF=population attributable fraction; AC=attributable cases; CI=confidence interval; LB=live births.

#### Tuberculosis

There were no recently published national TB prevalence surveys in the country. The Revised National Tuberculosis Control Programme (RNTCP) used a World Health Organization (WHO)-recommended approach to estimate prevalence. Briefly, in this approach data from two time points, that is, the 1956 National Prevalence Surveys and 2008 six District Prevalence Surveys, were pooled and individually weighted and adjustments applied for pediatric and extra-pulmonary TB, leading to an estimate of all age and all forms of TB prevalence for 2008. A constant rate of decline was then assumed and applied to arrive at estimates for the year 2011. Major limitations of this method include the convenient selection of districts, differences in screening methods, and assumptions regarding proportion of extra-pulmonary TB and decline rates. It was not possible to obtain a prevalence estimate for pulmonary TB; therefore, the reported all age and all forms of TB prevalence of 256 per 100,000 population was taken to be same as for pulmonary TB (22). This estimate was also not available by age, gender, or residence categories. On applying this rate to the age-specific national women population, we estimated the total number of TB cases to be 506,976 in rural and 252,361 in urban areas (Table 1). Meta-analysis of three studies provided a pooled OR of 2.33 (95% CI: 1.65, 3.28) for the association between TB and biomass fuel exposure (Supplementary Table 1), which in turn gave a PAF of 52% (95% CI: 35, 65) in rural and 20% (95% CI: 11, 30) in urban areas. In other words, about 0.3 (95% CI: 0.2, 0.4) out of 0.76 m total TB cases could be attributed to biomass cooking fuel exposure (Table 2 and Supplementary Table 3a and b).

#### Cataract

Since there were no national prevalence surveys of cataract, we used a single-site population-based survey conducted in 2002–03 among 614 women aged ≥50 years (23). Lens opacities were assessed using standard methods of lens photography and graded using the Lens Opacities Classification System (LOCS) II. The study provided age-specific prevalence of all cataract types. The prevalence was 54.5% in 50–59 year olds and 86.3% in 60–69 years olds. Estimates for age group ≥70 years were not included as the rates of cataract were almost universal (98%) among them and assumed to be mere consequences of the aging process. Another multicenter study (24) done in 2005–07 among about 2,800 women was also considered but not included because it included only those aged ≥60 years. However, its results were similar to that of the above study. By applying these rates to the corresponding age-specific national women population, we estimated the total number of cataract cases to be 35,476,487 in rural and 15,936,616 in urban areas (Table 1). Since cataract was not a rare condition among the study population, it violated the assumption of OR being approximately equal to RR. Therefore, OR calculated from the meta-analysis was converted to RR using a conversion formula given by Zhang and Yu (25). The formula used is as follows:

$$\text{RR}=\frac{\text{OR}}{\left(1-{\text{P}}_{\text{0}})+({\text{P}}_{\text{0}}\times \text{OR}\right)}$$

where RR is relative risk, OR is odds ratio, and Po is prevalence of disease in the unexposed population (assumed to be approximately equal to general population prevalence).

OR calculated from the meta-analysis was 2.16 (95% CI: 1.42, 3.26) (Supplementary Table 1), which was then converted to RR for the 50–59 age group which was 1.32 (95% CI: 1.16, 1.46) and for the 60–69 age group it was 1.08 (95% CI: 1.04, 1.11). The calculated PAF was 21% (95% CI: 12, 28) and 6% (95% CI: 3, 8) for the two age groups in rural areas and 6% (95% CI: 3, 8) and 1.0% (95% CI: 1, 2) for the two age groups in urban areas. The final number of cataract cases attributable to biomass cooking fuel exposure was 5.0 (95% CI: 2.8, 6.7) out of 51.4 m total cases (Table 2 and Supplementary Table 4a–e).

#### Stillbirth

National estimates of SBR were available for the year 2011 from the SRS report (20), which was 6 per 1,000 live births (LB) in both rural and urban areas. In order to calculate the total number of stillbirths, we needed the total number of LB, which was obtained by multiplying the CBR (per 1,000 population) for 2011 and the total population. CBR was given in the same SRS report, which was 23.3 for rural and 17.6 for rural areas (i.e. 19,342,347 rural and 6,605,834 urban births). The total number of stillbirths was calculated as 116,054 in rural and 39,635 in urban areas. A study by Cousens et al. (26) provided a much higher estimate for SBR at 22 per 1,000 LB, that is, total number of stillbirths were 425,532 in rural and 145,328 in urban areas. In order to account for the wide variation, we used both estimates (Table 1). Meta-analysis of two studies showed that the risk of stillbirth increased by 26% (OR = 1.26, 95% CI: 1.12, 1.43) among women exposed to biomass fuel (Supplementary Table 1). In rural areas, 18% (95% CI: 9, 26) of stillbirths and in urban areas 5% (95% CI: 2, 7) of stillbirths could be attributed to biomass fuel exposure, which translates to about 0.02 (95% CI: 0.01, 0.03) out of 0.15 m total cases at an SBR of 6 per 1,000 LB or 0.08 (95% CI: 0.04, 0.12) out of 0.57 m total cases at an SBR of 22 per 1,000 LB (Table 2 and Supplementary Table 5a–d).

## Discussion

The distribution of biomass fuel users is very socially polarized (28). Most users are poor, and live in rural and underserved areas of developing countries. Cooking with biomass fuel produces high levels of indoor air pollution with a range of health-damaging pollutants, including small soot particles that penetrate deep into the lungs. In poorly ventilated dwellings, toxicants can be several folds higher than acceptable levels. Exposure is particularly high among women and young children, who spend most of their time near the domestic stove. This study provides insights into burden of disease in the most vulnerable group, that is, women and in whom any intervention is likely to show the largest benefit. In simple terms, HAP from biomass fuel used for cooking is associated with 2.4 of 5.6 m cases of chronic bronchitis, 0.3 of 0.76 m cases of TB, 5.07 of 51.4 m cases of cataract among adult Indian women, and 0.02 of 0.15 m stillbirths across India.

### Biological plausibility

Several plausible biological mechanisms have been postulated for the association between these conditions and biomass fuel emissions. Some of them include oxidative stress, up-regulation of ribosome biogenesis (29), and DNA damage (30).

Toxicological studies in animals have suggested that acute exposure to smoke leads to pulmonary edema, perivascular infiltration of neutrophils, emphysematous alveolar destruction, and eosinophilia (31). A rat-based study found that rats exposed to wood smoke had elevated IL-4 levels in the broncho-alveolar lavage fluid and had more inflammatory lesions in the lungs (32).

A few researchers have reported that toxicants interfere with immune defense mechanisms by increasing cytotoxic T-cell and decreasing helper T-cell numbers, which in turn may be associated with increased risk of infections such as TB (33). Although evidence for detailed biological mechanisms of *Mycobacterium tuberculosis* activity and biomass fuel emission is limited, some indirect inferences can be drawn from studies done with tobacco smoke that has constituents similar to that of biomass fuel emissions. Particulate matter and chemicals found in smoke incite inflammation, impair the tracheobronchial mucociliary clearance system, and affect the functioning of pulmonary alveolar macrophages. These changes together might make the respiratory system defenseless against the TB bacilli, leading to infection and disease (34–37).

Several studies have indicated that biomass fuel smoke condensate enhance the formation of super-oxide radicals (38–40) increasing the risk of cataract, as does exposure to naphthalene and formaldehyde that are emitted during biomass fuel combustion (41, 42). Smoke from indoor air pollution can deplete antioxidants like plasma ascorbate, carotenoids, and glutathione and enhance formation of free radicals, which increase the risk of cataract (43–45). It has been reported by animal studies that firewood smoke condensate permeates the lens capsule, imparts color, and opacifies the lens in a dose-dependent manner (45).

Pollutants such as CO impair oxygen delivery to the fetus by forming carboxyhemoglobin in placento-fetal circulation, which may result in perinatal mortality and reduced birth weight (46). Ambient air PAH has been positively associated with the amount of PAH-DNA adducts in maternal and infant cord white blood cells, which in turn have been shown to significantly lower birth weight, birth length, and head circumference as compared to newborns with lesser adduct levels (47). Apart from these, several studies have reported associations between air pollution, cooking smoke, and adverse pregnancy outcomes such as perinatal mortality and low birth weight (13, 46, 47).

### Credibility of estimates

The strength of association found in our study between biomass fuel and chronic bronchitis is consistent with a meta-analysis done by Hu et al. (48). Biomass fuels have been considered one of the major causes of COPD burden in India (49, 50), which is in line with our study findings. Since chronic bronchitis represents just one of the components of COPD, the burden is likely to be much higher for the entire spectrum of COPD. With respect to TB, the calculations included all forms of TB, all age groups, both residence categories, and both genders; it is likely that the AC may be an overestimate. We know that prevalence of TB varies across all these categories (51, 52). Limitations in our understanding about age of onset of age-related cataract may have led us to underestimate those cases that occur prematurely before 50 years of age. Stillbirth is a condition, which is affected by several maternal and health access issues, thereby explaining the minimal role of biomass smoke in its burden as found in this study. Other research papers such as Lakshmi et al. (13) reported a PAF of 11% as compared to 5–18% in our study. Several assumptions made in the calculation of AC introduce uncertainties at each step; nevertheless the upper and lower limits give a rough sense of the burden due to each condition.

### Implications of estimates

The potentially avoidable burden due to a single risk factor namely biomass fuel has been explored here. This could be better understood in the context of alternative control strategies that are currently existent for these diseases. For chronic bronchitis/COPD, tobacco smoking is a major risk factor apart from ambient air pollution. The need for tobacco control in this country has been recently recognized and several population-level measures such as public awareness campaigns and legal sanctions are being implemented, but the sales of tobacco smoking products are ever on the rise. Therefore, current control measures for COPD include mainly early diagnosis, treatment, and disability limitation, which are only secondary- and tertiary-level preventive measures. In case of TB, major risk factors include poverty, close contact situations such as crowded households, and tobacco smoking. But the current control measures focus mainly on passive screening, early diagnosis, and treatment. The larger intervention of urban slum development and raising people’s standard of living would have to wait. The current strategy being followed for reducing cataract burden is again secondary- and tertiary-level preventive measures. Stillbirth prevention is accorded a lower priority among the objectives of maternal and child health programs, where the focus lies in prevention of maternal, neonatal, and infant deaths. All this being said, improving access to cleaner fuels and reducing HAP due to biomass fuel provide a single attractive primary prevention option to simultaneously control a range of disease conditions that is likely to provide long-term sustained reduction in disease burden. It is however important to note that AC do not equate to preventable cases because the various available interventions would have varying levels of effectiveness leading to differences in prevented cases.

## Strengths and limitations

Many studies that aim to estimate burden of disease concentrate on mortality, whereas this study has focused on morbidity in terms of AC. Also, burden estimates of four major diseases/conditions associated with biomass fuel exposure have been considered. There are several limitations in this analysis. In spite of our best efforts, it is possible that we might have missed some studies in the systematic review, but since our estimates are in line with other studies, this is less likely to be an issue. In the absence of reliable estimates, exposure to biomass cooking fuel exposure in adult Indian women was assumed to begin at 25 years of age, and the mean disease onset age was also set arbitrarily at 35 for chronic bronchitis, 25 for TB, and 50 for cataract. These assumptions implicitly incorporate the mean duration between exposure and disease onset. To our knowledge, this is the first study which uses epidemiologic evidence from Indian literature for estimating morbidity end points to generate evidence for public policy. Based on scientific research conducted in several Indian communities, we gather unequivocal evidence that high levels of air pollution, containing a mix of particulate and gaseous combustion products, produces adverse effects on population health at different stages of life and call for area-specific mitigation strategies.

Uncertainties result from gaps in domain knowledge especially as evidence of the health effects and assessment of exposure lies across scientific disciplines. Newer studies are required to provide information on 1) health effects to determine time of onset of disease; severity of disease; disease prevalence rates by age, gender, and residence; age-specific OR for disease outcomes; and estimates on household members and the primary cook – in to cook. In absence of age-specific OR and disease prevalence we have refrained from estimating disability adjusted life years (DALYs); 2) age-specific exposure rates, effect of intersubject variability, and effect of health status on risk; and 3) exposure misclassification, minimum threshold of exposure duration, concentration and characteristics of pollutants will help refine the estimates. In addition, personal air quality measurements over an extended period could help ascertain exposure more accurately; this remains largely unknown, in part because it has been difficult to quantify. Availability of a range of biomass fuels, varying composition of emissions and biomass emission factors of the same fuel vary across Indian states and in different seasons are some of the challenges in exposure assessment. Therefore, generalizing the health risk from exposure to different fuel sources under a single umbrella term may not be very representative, and may necessitate the need for obtaining region specific estimates. The extent of research on an environmental health issue in India depends upon the funding available and calls for systematic examination of health effects in the vulnerable population to year round high levels of pollutants. Determining the societal cost of disease and effect of disease severity would help direct public policy. Health cost assessment of different morbidity states would vary by place of residence but would help determine cost benefit of interventions.

One of the criticisms of PAF method is that adding up PAFs due to all risk factors may sum up to more than 100%. This is because of the inability to obtain the true association of a risk factor devoid of all confounding effects (53). Alternate methods such as Integrated Risk Function (54) available to evaluate PAF using a continuous exposure measure exist, but have their own merits and demerits.

## Recommendations

A WHO technical guidance recommends that member countries must achieve a 50% reduction in the usage of solid fuels in households by 2025. This is designed to strengthen action plans for prevention and control of NCD and move towards achieving the Millennium Development Goals (MDGs) (55).

In the past decade, there has been no significant indication of a decline in the trend of biomass fuel use (except for a minor decline in cow dung use) in rural India as shown by three major national surveys, namely the Census of 2001 (56), the third National Family Health Survey (NFHS) 2005–06 (57), and the NSSO 66th round 2009–10 (2), despite introduction of several government schemes to increase LPG access in rural areas. But these schemes do not seem to have made any major impact at the national level.

There are several challenges in the implementation of such schemes. Issues of acceptability, affordability, and poor awareness levels about alternate cooking fuels are major hurdles. On the supply side, pricing and distribution of different cooking fuels vary geographically and across demographic segments. Centralized planning without community participation affect the success of such schemes. They also overlook issues of program sustainability, quality, institutional strengthening, and community mobilization. In addition, area-specific challenges can occur, for example, arsenic exposure from cow dung combustion in arsenic-contaminated areas of the Ganga–Meghna–Brahmaputra (GMB) plain (58).

We believe that awareness generation among public and policymakers would help determine the path each community could take toward cleaner fuels. The path could include 1) wider access to LPG or Piped Natural Gas (PNG), and where appropriate smaller packaging and safe refilling options, 2) newer solar energy options, 3) availability of fuel-efficient biomass-based cook stoves, which can bring down exposure level close to cleaner fuels, and 4) awareness generation to increase adoption of simple household-level measures such as improved ventilation and selection of cleaner traditional fuels. A multifarious inclusive approach to achieve the common goal of universal clean fuel access should incorporate the local realities of each population segment (17).

Future studies can generate more accurate estimates of AC and calculate DALYs based on availability of new information and overcoming limitations stated here. Studies looking at the cost-effectiveness of promoting cleaner fuels as compared to competing preventive strategies will help policy makers to take informed decisions.

## Conclusions

We have drawn upon several research studies undertaken across India to determine estimates for a variety of health effects due to biomass cooking exposure. These estimates show that a large percentage of these health effects are avoidable. HAP due to biomass usage is a risk factor that affects all major sections of population that are of importance to public health, such as women, children, and elderly. It also influences disease burden in major categories such as maternal and child health, infectious diseases, NCD, and elderly health. Since women are the major sufferers, it also propagates gender-based health inequity. Biomass fuel, being a single factor, affects such a wide range of diseases and population groups. Therefore, it is an attractive proposition in terms of the benefits that could be derived from reducing or eliminating this single factor.

Changing usage of biomass-based cooking fuel depends on several factors, one of these is the cost of alternatives. Currently, Indian households receive an explicit subsidy of nearly 50% on domestic LPG, calculated through a price gap approach for the year 2013–14 (59); furthermore, close to 17% of national LPG consumption was imported in 2009–10 (60) and India imports almost four-fifths of its crude-oil needs (61). These, along with other factors determining the clean energy availability and access make the task of ‘universal access to clean fuel’ challenging. Nevertheless, incorporating the ‘universal access to clean fuel’ agenda within the broader framework of rural development and raising the standard of living will go a long way in reducing disease burden. Related ministries such as those of the new and renewable energy, health and family welfare, petroleum and natural gas, and rural development should adopt a coordinated approach to harness common good through joint efforts and reach maximum proportions of the affected. Reducing HAP will help achieve the MDGs, specifically those related to child mortality and improving maternal health.

## Supplementary Material

Disease burden due to biomass cooking-fuel-related household air pollution among women in India

Disease burden due to biomass cooking-fuel-related household air pollution among women in India

Disease burden due to biomass cooking-fuel-related household air pollution among women in India

Disease burden due to biomass cooking-fuel-related household air pollution among women in India

Disease burden due to biomass cooking-fuel-related household air pollution among women in India

Disease burden due to biomass cooking-fuel-related household air pollution among women in India

Disease burden due to biomass cooking-fuel-related household air pollution among women in India
