# Supplementary material for: Disease burden due to biomass cooking-fuel-related household air pollution among women in India
Source: Glob Health Action. 2014 Nov 4;7:10.3402/gha.v7.25326. doi: 10.3402/gha.v7.25326 (PMC4221659; doi:10.3402/gha.v7.25326)

Supplementary table 1. Meta-analysis for the association between biomass cooking fuel exposure and disease outcomes

Author	Study design	Location	Eligibility criteria	Exposure fuel	Comparison fuel	Sample size of women	Outcome	Reported OR (95% CI)	Variables adjusted	Pooled OR	I square, p value*
**Chronic bronchitis**											
Johnson P et al. (4)	Cross-sectional	Rural community based Tiruvallur	Non-smoking women, aged ≥30	Wood	LPG, electricity	900	COPD based on clinical examination, spirometry	1.43 (0.36, 5.73)	House type, kitchen type, passive smoking, cooking duration etc.,		
Sukhsohal ND et al. (27)	Cross-sectional	Rural community based Nagpur	Non-smoking non-pregnant women involved in cooking, aged ≥15	Biomass	LPG	760	PEFR less than 80% of the predicted was classified as abnormal lung function	2.49 (1.64, 3.77)	None	2.37 (1.59, 3.54)	0.0, 0.45
**Tuberculosis**											
Mishra VK et al. (9)	Secondary data analysis of NFHS-1	Country level, India	Women aged ≥20	Biomass (wood or dung)	Cleaner fuels (charcoal, coal, coke, lignite, kerosene, electricity, petroleum gas, or biogas)	1,23,824	Self-reported TB	2.74 (1.86, 4.05)	Separate kitchen, house type crowding, age, residence, education etc.,		
Behera D et al. (6)	Case-control	Hospital based, Delhi	Non-smoking women	Biomass	LPG	109 cases, 95 controls	Sputum positive TB	0.60 (0.20, 1.60)	Socio economic status, smoking in family, ventilation, exposure index, respiratory symptoms etc.,		
Lakshmi PVM et al. (7)	Case-control	Urban slums and rural areas,	Women aged 20 to 64 years without co-	Biomass	LPG	126 cases, 252 controls	Sputum positive TB	3.14 (1.15, 8.56)	Education, overcrowding, kitchen type, TB	2.33 (1.65, 3.28)	75.1, 0.01

		Chandigarh	morbid conditions						history, smoker in family etc.,		
**Cataract**											
Zodpey SP et al. (10)	Case-control	Hospital based, Nagpur	Women aged 35 to 75	Biomass (cow dung, wood)	LPG	223 cases, 223 controls	Lens opacity with visual acuity 6/60 or worse in affected eye, age related cataract without other causes	2.37 (1.44, 4.13)	Age, socio-economic status		
Pokhrel AK et al. (11)	Case-control	Hospital based, Indo-Nepal border	Women aged 35 to 75	Solid-fuel-burning stoves without flues	Kerosene, LPG, biogas, or electricity	206 cases, 203 controls	Ophthalmologist confirmed cataract cases without other causes	1.90 (1.00, 3.61)	Kitchen ventilation, working outsides, literacy, residence, source of light, incense burning	2.16 (1.42, 3.26)	0.0, 0.60
**Stillbirth**											
Mishra V et al. (14)	Secondary data analysis of NFHS-2	Country level, India	Ever-married women, aged 40 to 49 with complete birth histories	Biomass (wood, dung, or crop residues)	Cleaner fuels (electricity, LPG, biogas, or kerosene)	19,189	Delivery of a dead baby after the 28th week of pregnancy	1.44 (1.04, 1.97)	Tobacco smoke, woman's nutritional status, socioeconomic status, household conditions, residence etc.,		
Lakshmi PVM et al. (13)	Secondary data analysis of DLHS-2	Country level, India	Currently married aged 15 to 44	Wood	LPG, electricity	1,88,917	Delivery of a dead baby after the 28th week of pregnancy	1.24 (1.08, 3.27)	Literacy, lighting fuel, age at pregnancy, pregnancy related factors, SLI, residence, house type, religion, caste etc.,	1.26 (1.12, 1.43)	0.0, 0.39

*p value for Cochrane Q statistics

Supplementary table 1a. Reasons for exclusion of studies from meta-analysis

Author	Study design	Study location (rural/urban)	Age group (years)	Exposure cooking fuel	Comparison cooking fuel	Disease Definition	Sample size	Odds ratio (95% CI)	Reasons for exclusion
**Cataract**									
Mishra VK et al. (12)	National survey	Urban, Rural	≥30	Biomass	Coal, kerosene, electricity, LPG, bio gas	Self -reported partial blindness/cataract, complete blindness	173520	1.12 (1.3 to 1.50)	OR for blindness (partial or complete)
Mohan M et al. (63)	Case-control	Urban, hospital based	37-62	Cow dung, wood	LPG	Physician determined	Cases - 1441 Control - 549	0.62 (0.40 to 0.98)	OR not available for women
Saha A et al. (64)	Cross sectional			Wood	LPG	Physician determined	469	3.47 (1.05 to 11.50)	OR not available for women
**TB**									
Kolappan C et al. (8)	Case-control	Urban, Rural hospital based	15 to 60	Biomass	Cleaner fuel	Chest radiograph, sputum smear or culture examination	Cases - 255 Controls - 1275	1.7 (1.0 to 2.9)	OR not available for women
Shetty N et al. (65)	Case-control	Urban, hospital based	15-83	Biomass	Gas, electricity	Sputum smear test and chest X-ray	185 Cases and Controls	23 (3.1 to 170.3)	Small sample size, wide age
**COPD**									
Qureshi KA (66)	Cross sectional	Rural	15-50	Solid cooking fuel	Different duration of exposure	FVC, FEV1 tests	138	2.10 (1.5 to 2.94)	Use of solid fuel for heating and burning
Jain NK et al. (67)	Not specified	Urban	Mean age male 61.57, Female 58.34	Biomass	Non smokers	Spirometric evaluation, chest X-ray, CT scan	702	Prevalence of COPD	OR not included

Author	Study design	Study location (rural/urban)	Age group (years)	Exposure cooking fuel	Comparison cooking fuel	Disease Definition	Sample size	Odds ratio (95% CI)	Reasons for exclusion
Dutt D et al. (68)	Cross sectional	Urban slum	15-60	Biofuels	Kerosene, LPG	Pulmonary function test, respiratory morbidity	315 (97 biomass, 100 kerosene, 98 LPG) women	4.17 (0.46 to 38.02)	Small sample size, wide age group
Jindal SK et al. (69)	Cross-sectional	Rural and Urban	≥35	Solid fuel	No cooking	Questionnaire	35295	0.635 (0.524 to 0.771)	OR not given for women exclusively, comparison with no cooking
Behera D et al. (70)	Cross-sectional	Rural	Not specified	Biomass	LPG, kerosene, mixed	Questionnaire, spirometry	3701	Prevalence	OR not included.
Mahesh PA et al. (71)	Cross-sectional	Rural	>30	Biomass fuel	Non-smokers	Questionnaire	3953	Prevalence rate mentioned	OR not given
**Stillbirths**									
Wylie BJ et al. (72)	Cohort	Urban, rural facility	>15	Wood, cow dung, charcoal	Gas	Questionnaire	1744	4% vs 0%	Small sample size
Tielsch JM et al. (15)	Cohort	Rural	Live births to 6 months	Wood, dung	Biogas or kerosene	Birth weight, Small for gestational age	11728 live births	SGA 1.21 (1.11 to 1.31)	No OR for still birth
Mavalankar DV et al. (62)	Case control	Urban, hospital based	30-34	Cooking smoke	Not specified		451 still births 1465 controls	1.5 (1.0 to 2.1)	Not adjusted for second hand smoke

Supplementary table 1b. Forest Plot for studies included in meta-analysis

Outcome	Estimate	Lower CI	Upper CI	Reference
Chronic bronchitis	1.43	0.36	5.73	Johnson P, 2012
Chronic bronchitis	2.49	1.64	3.77	Sukhsohal ND, 2013
Chronic bronchitis	2.37	1.59	3.54	Pooled OR
TB	0.60	0.20	1.60	Behera D, 2010
TB	3.14	1.15	8.56	Lakshmi PVM,
TB	2.74	1.86	4.05	Mishra VK, 1999
TB	2.33	1.65	3.28	Pooled OR
Cataract	2.37	1.44	3.61	Zodpey SP, 1999
Cataract	1.90	1.00	3.61	Pokhrel AK, 2005
Cataract	2.16	1.42	3.26	Pooled OR
Stillbirth	1.24	1.08	3.27	Lakshmi PVM, 2013
Stillbirth	1.44	1.04	1.97	Mishra V, 2005
Stillbirth	1.26	1.12	1.43	Pooled OR

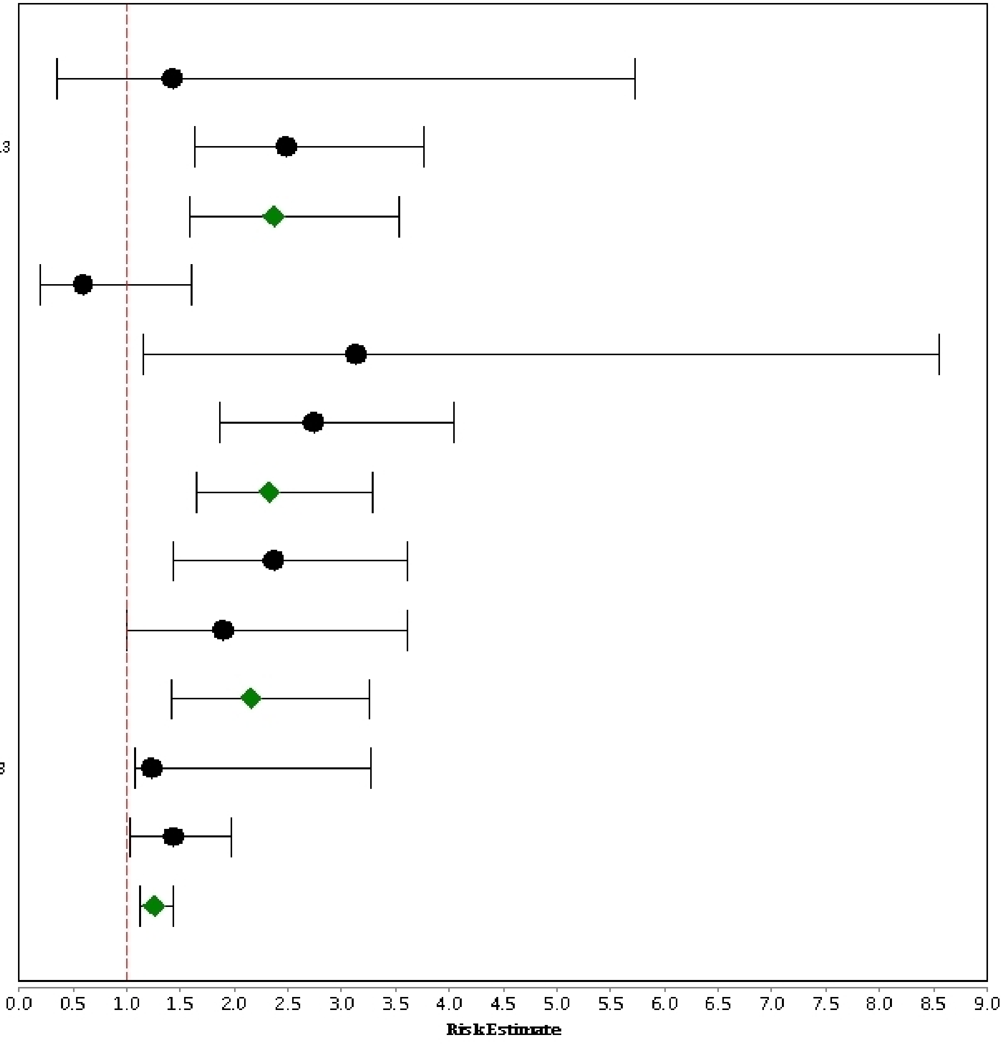

Supplement: Disease burden due to biomass cooking-fuel-related household air pollution among women in India [file GHA-7-25326-s002.pdf]
