# Supplementary material for: Disease burden due to biomass cooking-fuel-related household air pollution among women in India
Source: Glob Health Action. 2014 Nov 4;7:10.3402/gha.v7.25326. doi: 10.3402/gha.v7.25326 (PMC4221659; doi:10.3402/gha.v7.25326)
Supplement: Disease burden due to biomass cooking-fuel-related household air pollution among women in India [file GHA-7-25326-s003.pdf]

**Supplementary table 2**. Estimates for chronic bronchitis

| Urban | | | | | | | | | | | | | | |
|---|---|---|---|---|---|---|---|---|---|---|---|---|---|---|
| age group (years) | total no. urban_females census | pe - proportion of biomass exposure in urban women | Population exposed - total no. of females exposed to Biomass | OR | | | PAF (urban) | | | Rate (unexposed) - prevalence of chronic bronchitis (Jindal et al.. 2012 (3)) | total cases in population | AC | | |
| | | | | Est | lowerCI | UpperCI | Est | lowerCI | UpperCI | | | Est | lowerCI | UpperCI |
| 35-44 | 26,161,110 | 0.1880 | 4,918,289 | 2.37 | 1.59 | 3.54 | 0.20 | 0.10 | 0.32 | 0.0133 | 347,698 | 71,212 | 34,716 | 112,373 |
| 45-54 | 18,340,002 | 0.1880 | 3,447,920 | 2.37 | 1.59 | 3.54 | 0.20 | 0.10 | 0.32 | 0.0255 | 468,400 | 95,933 | 46,767 | 151,382 |
| 55-64 | 11,967,363 | 0.1880 | 2,249,864 | 2.37 | 1.59 | 3.54 | 0.20 | 0.10 | 0.32 | 0.0343 | 410,982 | 84,173 | 41,035 | 132,825 |
| 65-74 | 6,530,620 | 0.1880 | 1,227,757 | 2.37 | 1.59 | 3.54 | 0.20 | 0.10 | 0.32 | 0.0542 | 353,633 | 72,427 | 35,309 | 114,291 |
| >=75 | 3,245,269 | 0.1880 | 610,111 | 2.37 | 1.59 | 3.54 | 0.20 | 0.10 | 0.32 | 0.0625 | 202,948 | 41,566 | 20,263 | 65,591 |
| | | | | | | | | | | total | 1,783,660 | 365,310 | 178,090 | 576,462 |
| **Rural** | | | | | | | | | | | | | | |
| 35-44 | 50,937,056 | 0.8260 | 42,074,008 | 2.37 | 1.59 | 3.54 | 0.53 | 0.33 | 0.68 | 0.0133 | 676,986 | 359,394 | 221,820 | 458,465 |
| 45-54 | 35,054,663 | 0.8260 | 28,955,152 | 2.37 | 1.59 | 3.54 | 0.53 | 0.33 | 0.68 | 0.0255 | 895,288 | 475,285 | 293,349 | 606,303 |
| 55-64 | 26,678,850 | 0.8260 | 22,036,730 | 2.37 | 1.59 | 3.54 | 0.53 | 0.33 | 0.68 | 0.0343 | 916,202 | 486,387 | 300,202 | 620,466 |
| 65-74 | 16,535,168 | 0.8260 | 13,658,049 | 2.37 | 1.59 | 3.54 | 0.53 | 0.33 | 0.68 | 0.0542 | 895,380 | 475,333 | 293,379 | 606,365 |
| >=75 | 7,184,673 | 0.8260 | 5,934,540 | 2.37 | 1.59 | 3.54 | 0.53 | 0.33 | 0.68 | 0.0625 | 449,305 | 238,524 | 147,219 | 304,276 |
| | | | | | | | | | | total | 3,833,161 | 2,034,922 | 1,255,969 | 2,595,875 |
| | | | | | | | | | | grand total | 5,616,821 | 2,400,233 | 1,434,059 | 3,172,337 |
