# Supplementary material for: Disease burden due to biomass cooking-fuel-related household air pollution among women in India
Source: Glob Health Action. 2014 Nov 4;7:10.3402/gha.v7.25326. doi: 10.3402/gha.v7.25326 (PMC4221659; doi:10.3402/gha.v7.25326)
Supplement: Disease burden due to biomass cooking-fuel-related household air pollution among women in India [file GHA-7-25326-s004.pdf]

Supplementary table 3a. Estimates for TB (Urban)

age group (years)	total no. urban_females census	pe - proportion of biomass exposure in urban women	Population exposed - total no. urban females exposed to Biomass	OR			PAF (urban)			Rate (unexposed) - prevalence of chronic bronchitis (Jindal et al.. 2012 (3))	total cases in population	AC		
				Est	lowerCI	UpperCI	Est	lowerCI	UpperCI			Est	lowerCI	UpperCI
35-44	26,161,110	0.1880	4,918,289	2.37	1.59	3.54	0.20	0.10	0.32	0.0133	347,698	71,212	34,716	112,373
45-54	18,340,002	0.1880	3,447,920	2.37	1.59	3.54	0.20	0.10	0.32	0.0255	468,400	95,933	46,767	151,382
55-64	11,967,363	0.1880	2,249,864	2.37	1.59	3.54	0.20	0.10	0.32	0.0343	410,982	84,173	41,035	132,825
65-74	6,530,620	0.1880	1,227,757	2.37	1.59	3.54	0.20	0.10	0.32	0.0542	353,633	72,427	35,309	114,291
>=75	3,245,269	0.1880	610,111	2.37	1.59	3.54	0.20	0.10	0.32	0.0625	202,948	41,566	20,263	65,591
										total	1,783,660	365,310	178,090	576,462

Supplementary table 3b. Estimates for TB (Rural)

age group (years)	total no. rural_female census	pe - proportion of biomass exposure in rural women	Population exposed - total no. rural females exp to Biomass	OR			PAF (rural)			Rate (unexposed) - prevalence of TB (annual report (22))	total cases in population	AC		
				Est	lowerCI	UpperCI	Est	lowerCI	UpperCI			Est	lowerCI	UpperCI
25-34	61,647,268	0.8260	50,920,643	2.33	1.65	3.28	0.52	0.35	0.65	0.00256	157,817	82,615	55,132	103,082
35-44	50,937,056	0.8260	42,074,008	2.33	1.65	3.28	0.52	0.35	0.65	0.00256	130,399	68,262	45,553	85,173
45-54	35,054,663	0.8260	28,955,152	2.33	1.65	3.28	0.52	0.35	0.65	0.00256	89,740	46,978	31,350	58,616
55-64	26,678,850	0.8260	22,036,730	2.33	1.65	3.28	0.52	0.35	0.65	0.00256	68,298	35,753	23,859	44,610
65-74	16,535,168	0.8260	13,658,049	2.33	1.65	3.28	0.52	0.35	0.65	0.00256	42,330	22,159	14,788	27,649
>=75	7,184,673	0.8260	5,934,540	2.33	1.65	3.28	0.52	0.35	0.65	0.00256	18,393	9,628	6,425	12,014
										total	506,976	265,396	177,107	331,143
										grand total	759,337	315,874	204,587	406,860
