# Supplementary material for: Disease burden due to biomass cooking-fuel-related household air pollution among women in India
Source: Glob Health Action. 2014 Nov 4;7:10.3402/gha.v7.25326. doi: 10.3402/gha.v7.25326 (PMC4221659; doi:10.3402/gha.v7.25326)
Supplement: Disease burden due to biomass cooking-fuel-related household air pollution among women in India [file GHA-7-25326-s005.pdf]

Supplementary table 4a. Estimates for cataract

age group (years)	total no. urban_females census	pe - proportion of biomass exposure in urban women	Population exposed - total no. urban females exposed to Biomass	OR			PAF (urban)			Rate (unexposed) - prevalence cataract (Murthy et al.. (23))	total cases in population	AC		
				Est	lowerCI	UpperCI	Est	lowerCI	UpperCI			Est	lowerCI	UpperCI
50-59	14,348,805	0.1880	2,697,575	2.16	1.42	3.26	0.18	0.07	0.30	0.545	7,820,099	1,400,078	572,287	2,331,848
60-69	9,405,003	0.1880	1,768,141	2.16	1.42	3.26	0.18	0.07	0.30	0.863	8,116,518	1,453,148	593,980	2,420,236
										total	15,936,616	2,853,226	1,166,267	4,752,084

Supplementary table 4b Cataract based on OR

age group (years)	total no. rural_female census	pe - proportion of biomass exposure in rural women	Population exposed - total no. rural females exposed to Biomass	OR			PAF (rural)			Rate (unexposed) - prevalence cataract (Murthy et al.. 2007 (23))	total cases in population	AC		
				Est	lowerCI	UpperCI	Est	lowerCI	UpperCI			Est	lowerCI	UpperCI
50-59	28,567,226	0.8260	14,477,576	2.16	1.42	3.26	0.49	0.26	0.65	0.545	15,569,138	7,618,236	4,010,071	10,138,220
60-69	23,067,612	0.8260	22,036,730	2.16	1.42	3.26	0.49	0.26	0.65	0.863	19,907,349	9,740,994	5,127,445	12,963,151
										total	35,476,487	17,359,231	9,137,516	23,101,371
										grand total	51,413,104	20,212,456	10,303,783	27,853,455

Supplementary table 4c

age group (years)	total no. urban_females census	pe - proportion of biomass exposure in urban women	Population exposed - total no. urban females exposed to Biomass	RR			PAF (urban)			Rate (unexposed) - prevalence cataract (Murthy et al.. 2007 (23))	total cases in population	AC		
				Est	lowerCI	UpperCI	Est	lowerCI	UpperCI			Est	lowerCI	UpperCI
50-59	14,348,805	0.1880	1,723,960	1.32	1.16	1.46	0.06	0.03	0.08	0.545	7,820,099	448,162	226,287	624,546
60-69	9,405,003	0.1880	2,249,864	1.08	1.04	1.11	0.015	0.008	0.019	0.863	8,116,518	119,400	65,036	157,264
										total	15,936,616	567,563	291,323	781,810

Supplementary table 4d Cataract based on RR

age group (years)	total no. rural_female census	pe - proportion of biomass exposure in rural women	Population exposed - total no. rural females exposed to Biomass	RR*			PAF (rural)			Rate (unexposed) - prevalence cataract (Murthy et al.. 2007 (23))	total cases in population	AC		
				Est	lowerCI	UpperCI	Est	lowerCI	UpperCI			Est	Lower CI	Upper CI
50-59	28,567,226	0.8260	14,477,576	1.32	1.16	1.46	0.21	0.12	0.28	0.545	15,569,138	3,281,930	1,802,405	4,298,169
60-69	23,067,612	0.8260	22,036,730	1.08	1.04	1.11	0.06	0.03	0.08	0.863	19,907,349	1,225,502	682,293	1,590,153
										total	35,476,487	4,507,432	2,484,698	5,888,322
										grand total	51,413,104	5,074,994	2,776,021	6,670,132

*Please note that RR are different for the age strata as prevalence of disease is different for each age group, PAF will also be different across strata

Supplementary table 4e Cataract based on estimated RR

Age group	Cataract ESTIMATED RR = (OR)/ (1-Pe)+(Pe X OR); where Pe is the prevalence of disease in unexposed		
	RR		
	Est	Lower CI	Upper CI
50-59	1.32	1.16	1.46
60-69	1.08	1.04	1.10
	Please note that OR and RR are different for the age strata		
