# Supplementary material for: Disease burden due to biomass cooking-fuel-related household air pollution among women in India
Source: Glob Health Action. 2014 Nov 4;7:10.3402/gha.v7.25326. doi: 10.3402/gha.v7.25326 (PMC4221659; doi:10.3402/gha.v7.25326)
Supplement: Disease burden due to biomass cooking-fuel-related household air pollution among women in India [file GHA-7-25326-s006.pdf]

**Supplementary table 5a**. Estimates for stillbirth

| | | | | | | | OR | | | PAF (urban) | | | AC | | |
|---|---|---|---|---|---|---|---|---|---|---|---|---|---|---|---|
| total urban population census | birth rate/ population (urban) | still birth rate/live birth (20) | total births in population | total still births in population | pe - proportion of biomass exposure in urban women | total no. urban population exposed to Biomass | Est | Lower CI | Upper CI | Est | Lower CI | Upper CI | Est | Lower CI | Upper CI |
| 375,331,461 | 0.0176 | 0.006 | 6,605,834 | 39,635 | 0.1880 | 70,562,315 | 1.26 | 1.12 | 1.43 | 0.05 | 0.02 | 0.07 | 1,847 | 874 | 2,964 |

**Supplementary table 5b**

| | | | | | | | | | OR | | | PAF (rural) | | | AC | | |
|---|---|---|---|---|---|---|---|---|---|---|---|---|---|---|---|---|---|
| total rural population census | birth rate/ population (rural) | total no. of births in exp (rural) | still birth rate/live birth (20) | total births in population | total still births in population | total no. of still births in exp (rural) | pe - proportion of biomass exposure in rural women | total no. rural population exposed to Biomass | Est | Lower CI | Upper CI | Est | Lower CI | Upper CI | Est | Lower CI | Upper CI |
| 830,143,635 | 0.023 | 15,839,639 | 0.006 | 19,342,347 | 116,054 | 110,877 | 0.8260 | 685,698,643 | 1.26 | 1.12 | 1.43 | 0.18 | 0.09 | 0.26 | 20,517 | 10,466 | 30,417 |
| | | | grand total | 25,948,180 | 155,689 | 119,472 | | | | | | | | | 22,365 | 11,340 | 33,381 |

**Supplementary table 5c**

| | | | | | | | | | OR | | | PAF (urban) | | | AC | | |
|---|---|---|---|---|---|---|---|---|---|---|---|---|---|---|---|---|---|
| total urban population census | birth rate/population (urban) | total no. of births in exp (urban) | still birth rate/live birth (Cousens S et al.. 2011 (26)) | total births in population | total still births in population | total no. of still births in exp (urban) | pe - proportion of biomass exposure in urban women | total no. urban population exposed to Biomass | Est | Lower CI | Upper CI | Est | Lower CI | Upper CI | Est | lower CI | Upper CI |
| 375,331,461 | 0.018 | 1,227,784 | 0.022 | 6,605,834 | 145,328 | 27,011 | 0.1880 | 70,562,315 | 1.26 | 1.12 | 1.43 | 0.05 | 0.02 | 0.07 | 6,773 | 3,206 | 10,870 |

## Supplementary table 5d

| | | | | | | | | | OR | | | PAF (rural) | | | AC | | |
|---|---|---|---|---|---|---|---|---|---|---|---|---|---|---|---|---|---|
| total rural population census | birth rate/population (rural) | total no. of births in exp (rural) | still birth rate/live birth (Cousens S et al.. 2011 (26)) | total births in population | total still births in population | total no. of still births in exp (rural) | pe - proportion of biomass exposure in rural women | total no. rural population exposed to Biomass | Est | Lower CI | Upper CI | Est | Lower CI | Upper CI | Est | Lower CI | Upper CI |
| 830,143,635 | 0.023 | 15,839,639 | 0.022 | 19,342,347 | 425,532 | 348,472 | 0.8260 | 685,698,643 | 1.26 | 1.12 | 1.43 | 0.18 | 0.09 | 0.26 | 75,231 | 38,375 | 111,528 |
| | | | grand total | 25,948,180 | 570,860 | 375,483 | | | | | | | | grand total | 82,003 | 41,581 | 122,397 |
