# Supplementary material for: Disease burden due to biomass cooking-fuel-related household air pollution among women in India
Source: Glob Health Action. 2014 Nov 4;7:10.3402/gha.v7.25326. doi: 10.3402/gha.v7.25326 (PMC4221659; doi:10.3402/gha.v7.25326)
Supplement: Disease burden due to biomass cooking-fuel-related household air pollution among women in India [file GHA-7-25326-s007.pdf]

Supplementary text

Methodology of systematic review and meta-analysis

General considerations

Two authors independently conducted literature search using predefined inclusion and exclusion criteria. An attempt was made to retrieve all published literature including journal articles, conference presentations, government documents and unpublished literature through emails.

Supplementary box 1 Study selection criteria

**A)**	**Inclusion criteria**
**1**	Articles in English, or those having detailed summary in English.
**2**	No time restriction.
**3**	Primary research or secondary data analysis, case-control, cohort or cross-sectional data, systematic review, meta-analysis at community or hospital level with essential data for calculating OR or RR.
**4**	Study population was adult women who were primary cooks, preferably non-smokers
**5**	Studies that provided information for age groups ≥18 years and either sex.
**6**	Measure of association – Odds ratio or relative risk restricted or stratified or adjusted for confounders, mainly tobacco smoking and provided for female gender
**7**	Exposure group – Cooking fuel used was either wood, crop residue or cow dung.
**8**	Control group – Cooking fuel used was either LPG, biogas, kerosene or electricity.
**9**	Outcome – Any one of the four study conditions (chronic bronchitis, tuberculosis, cataract, still births)
**10**	Setting – India, if not available then South-East Asia
**B)**	**Exclusion criteria**
	Studies exclusively including use of solid fuel for area heating, or coal as cooking fuel
	Studies including males
